# The Temporal Evolution and Global Spread of *Cauliflower mosaic virus*, a Plant Pararetrovirus

**DOI:** 10.1371/journal.pone.0085641

**Published:** 2014-01-21

**Authors:** Ryosuke Yasaka, Huy D. Nguyen, Simon Y. W. Ho, Sebastián Duchêne, Savas Korkmaz, Nikolaos Katis, Hideki Takahashi, Adrian J. Gibbs, Kazusato Ohshima

**Affiliations:** 1 Laboratory of Plant Virology, Faculty of Agriculture, Saga University, Saga, Japan; 2 The United Graduate School of Agricultural Sciences, Kagoshima University, Kagoshima, Japan; 3 School of Biological Sciences, University of Sydney, Sydney, New South Wales, Australia; 4 Department of Plant Protection, Faculty of Agriculture, University of Canakkale Onsekiz Mart, Canakkale, Turkey; 5 Plant Pathology Laboratory, Faculty of Agriculture, Aristotle University of Thessaloniki, Thessaloniki, Greece; 6 Graduate School of Agricultural Science, Faculty of Agriculture, Tohoku University, Sendai, Japan; 7 Emeritus Faculty, Australian National University, Canberra, Australia; Institute of Infectious Disease and Molecular Medicine, South Africa

## Abstract

*Cauliflower mosaic virus* (CaMV) is a plant pararetrovirus with a double-stranded DNA genome. It is the type member of the genus *Caulimovirus* in the family *Caulimoviridae*. CaMV is transmitted by sap inoculation and in nature by aphids in a semi-persistent manner. To investigate the patterns and timescale of CaMV migration and evolution, we sequenced and analyzed the genomes of 67 isolates of CaMV collected mostly in Greece, Iran, Turkey, and Japan together with nine published sequences. We identified the open-reading frames (ORFs) in the genomes and inferred their phylogeny. After removing recombinant sequences, we estimated the substitution rates, divergence times, and phylogeographic patterns of the virus populations. We found that recombination has been a common feature of CaMV evolution, and that ORFs I–V have a different evolutionary history from ORF VI. The ORFs have evolved at rates between 1.71 and 5.81×10^−4^ substitutions/site/year, similar to those of viruses with RNA or ssDNA genomes. We found four geographically confined lineages. CaMV probably spread from a single population to other parts of the world around 400–500 years ago, and is now widely distributed among Eurasian countries. Our results revealed evidence of frequent gene flow between populations in Turkey and those of its neighboring countries, with similar patterns observed for Japan and the USA. Our study represents the first report on the spatial and temporal spread of a plant pararetrovirus.

## Introduction

Studies of the population genetics of plant viruses are important for understanding the evolution of virus-host interactions [1]–[3], because plant viruses sometimes adapt rapidly to new or resistant hosts [4]–[6]. Most evolutionary studies of plant viruses have focused on those with single-stranded RNA (ssRNA) genomes [3], [7]–[9], partly because many plant viruses have such genomes. Another reason for this focus is that they have error-prone RNA polymerases, and therefore evolve at a measurable rate which complicates the creation of resistant plant cultivars. Populations of plant viruses with single-stranded DNA (ssDNA) genomes have also been studied, including those of begomoviruses and mastreviruses in the family *Geminiviridae*, which also evolve at a measurable rate, are emergent viruses and damage many crops worldwide [10]–[15]. These reports showed that virus populations have been shaped by selection, founder effects, and recombination. On the other hand, there has been little work on the population genetics of plant viruses with double-stranded DNA (dsDNA) genomes.

*Cauliflower mosaic virus* (CaMV) has a dsDNA genome and is the type species of the genus *Caulimovirus* in the family *Caulimoviridae* [16]. Although it infects plants, CaMV is grouped with the hepadnaviruses of animals as a pararetrovirus because it has icosahedral virions and because its replication strategy involves an RNA intermediate [16]. CaMV is transmitted by sap inoculation, and in nature by aphids such as *Brevicoryne brassicae*, *Myzus persicae*, and at least 25 other species in a semi-persistent manner. CaMV reduces the yield and quality of brassica crops worldwide. In nature, its host range seems to be limited to plants of the family Brassicaceae, but some isolates are able to infect plants of the family Solanaceae experimentally [17].

The genome of CaMV is a circular dsDNA molecule of about 8000 nt with three short single-stranded regions: two in one strand, one in the other [18]. It has seven open reading frames (ORFs) and large and small intergenic regions [16]. Located between ORF VI and ORF I, the large intergenic region contains the pregenomic RNA 35S promoter, the RNA polyadenylation signal, and the minus-strand primer-binding site. The small intergenic region, containing the 19S promoter, is located between ORFs V and VI. The genome encodes six viral gene products that have been detected *in planta*. Protein P1 is the cell-to-cell movement protein, P2 is the aphid transmission factor, P3 is the virion-associated aphid transmission factor, P4 is the coat protein precursor, P5 is the polyprotein precursor of proteinase, reverse transcriptase, and ribonuclease, and P6 is the translation transactivator/viral silencing suppressor and also the major protein of the inclusion body matrix [19]. No protein encoded by ORF VII has been detected *in planta* and the function of this ORF is still unknown [20].

The possibility of controlling a pathogen is improved if we know when, where, and how it first became established in the host population of interest, namely its ‘centre of emergence’. This is analogous to the ‘centre of diversity’ of crop species [21]. Brassicas are major vegetable crops for human and animal consumption worldwide; most cultivars originated in South-West Eurasian countries [22]–[26]. Many plant viruses infect these crops, with *Turnip mosaic virus* (TuMV), *Cucumber mosaic virus* (CMV), and CaMV being particularly well known. We assessed the population structure of TuMV in a number of previous studies [9], [27]–[29].

Only nine full nucleotide sequences of CaMV genomes have been reported so far [30], providing insufficient data to characterize the population structure of the virus. Here, we report the genomic sequences of CaMV isolates from brassica hosts in the Eurasian region. We analyzed our 69 new sequences in combination with nine published sequences to estimate the phylogeny, the evolutionary timescale, and the degree of divergence between populations in different countries. Our analyses provide insights into the spatial and temporal evolution of several CaMV populations.

## Materials and Methods

### Virus isolates and host tests

We surveyed the brassica crop-producing areas of Greece, Iran, Turkey, and Japan during the growing seasons of 2001–2010. All collected samples were tested by direct double-antibody sandwich enzyme-linked immunosorbent assay (DAS-ELISA) [31] using the antiserum to CaMV (BIOREBA, Switzerland). Some of the Japanese isolates were gifts from NIAS Genebank, Japan, whereas the remaining isolates were collected from private gardens and fields, with permission from owners. No specific permissions were required for the locations/activities. Our field studies did not involve endangered or protected species. Details of the CaMV isolates, their place of origin, original or common (English) host plant, year of isolation, and host type are shown in Table S1 in File S1.

All of the isolates were sap-inoculated to the Japanese *Brassica rapa* cv. Hakatasuwari plants and serially cloned through single lesions at least three times using chlorotic local lesions that appeared approximately 10 days after the inoculation. The biological cloning step is important because CaMV was often in mixed infections with TuMV and/or CMV, and some plants contained a mixture of CaMV strains (data not shown). Hence, there is a possibility that artificial recombination events will be detected in the sequence data. Purified CaMV isolates were propagated in *Brassica rapa* cv. Hakatasuwari plants. Plants infected systemically with each of the CaMV isolates were homogenized in 0.01 M potassium phosphate buffer (pH 7.0) and mechanically inoculated onto young Japanese cultivars of *B. rapa* cv. Hakatasuwari, and *Raphanus sativus* cvs. Akimasari-2go, Taibyo-sobutori and Everest, *B. oleracea* var. *capitata* cvs. Shinsei, Ryosan 2go and Soushu, *B. oleracea* var. *botrytis* cv. Snow queen, *B. oleracea* var. *italica* cv. Challenger, *B. oleracea* var. *Gongylodes* cv. Grand duke, *B. napus* cv. Otsubu, *B. pekinensis* cv. Nozaki 1-go, and *B. campestris* var. *Narinosa* cv. Tatsuai. Inoculated plants were kept for at least four weeks in a glasshouse at Saga University at 25°C.

### Viral DNA and sequence data

Viral DNAs were extracted from CaMV-infected *B. rapa* leaves using DNeasy Plant Mini Kit (Qiagen K.K., Japan). The DNAs were amplified using high-fidelity Platinum *Pfx* DNA polymerase (Invitrogen, Japan). The PCR products were separated by electrophoresis in agarose gels and purified using a QIAquick Gel Extraction kit (Qiagen K.K., Japan). Sequences from each isolate were determined using at least three overlapping independent PCR products to cover the complete genome. The sequences of the PCR products or cloned fragments of adjacent regions of the genome overlapped by at least 300 nt to ensure that they were from the same genome and were not from different components of a genome mixture. Each PCR product was sequenced by primer walking in both directions using a BigDye Terminator v3.1 Cycle Sequencing Ready Reaction kit (Life Technologies, Japan) and an Applied Biosystems Genetic Analyzer DNA model 310. Ambiguous nucleotides in any sequence were checked in sequences obtained from at least three to five other independent plasmids, which were cloned into *Eco*R V site of plasmid pZErO-2. Sequence data were assembled using BioEdit v5.0.9 [32].

The genomic sequences of the 76 isolates were used for a range of evolutionary analyses. The genomic sequence of ID1 isolate of *Horseradish latent virus* (HRLV, Accession code NC_018858) was used as an outgroup because BLAST searches showed that it was most closely and consistently related to those of CaMV. We aligned all genes via the corresponding amino acid sequences using CLUSTAL X2 [33] with TRANSALIGN (kindly supplied by Georg Weiller, Australian National University). ORF I to ORF V sequences were then reassembled to form concatenated ORF I–V sequences of 5,106 nt. We discarded overlapping sequences between ORF III and ORF IV (9 nt) and between ORF IV and ORF V (23 nt). The aligned ORF VI sequences were 1,554 nt in length.

### Recombination analyses

We investigated recombination in the genomic sequences using RDP [34], GENECONV [35], BOOTSCAN [36], MAXCHI [37], CHIMAERA [38], and SISCAN [39], all implemented in RDP4 [40]. We also analyzed the data using the original PHYLPRO [41], SISCAN version 2 [39]. These analyses were done using default settings for the different detection programs and a Bonferroni-corrected *P*-value cut-off of 0.01, and overlapping 100- and 50-nt slices. These analyses also assessed which non-recombinant sequences contained regions that were most closely related to those of the recombinant sequences, indicating the lineages that most likely provided those regions of the recombinant genomes. For simplicity, we refer to these as the ‘parental isolates’ of recombinants. To examine the impact of gaps introduced when aligning the CaMV sequences to the outgroup, we checked for evidence of recombination after aligning the CaMV with the outgroup excluded. Finally, the aligned sequences were analyzed for recombination using the Recombination Analysis Tool (RAT) [42]. This analysis compared the percentage of nucleotide similarities using a sliding window of 30 nt, allowing detection of breakpoints among sequences.

We included the recombinant genomes in our analyses of individual ORFs when there was no evidence of within-ORF recombination, but discarded recombinant genomes for our phylogenetic estimates of rates and timescales. Moreover, we discarded 192 nt and 93 nt of the 5′ and 3′ ends, respectively, from the aligned ORF VI 1,554 nt. Specifically, we discarded both of the ends from the major recombination sites that were found, and used 1,269 nt for the subsequent ORF VI analyses.

### Estimation of substitution rates and divergence times

The phylogenetic relationships of the sequences and of their constituent ORFs were estimated using the Neighbor-Net method in SPLITSTREE v4.11.3 [43], and using maximum likelihood in PhyML v3 [44]. For the maximum-likelihood (ML) analysis, we used the general time-reversible (GTR) model of nucleotide substitution, with rate variation among sites modelled using a gamma distribution and a proportion of invariable sites (GTR+Γ_4_+I). This model was selected in R [45] using the Bayesian information criterion, which has been shown to perform well in a variety of scenarios [46]. Branch support was evaluated by bootstrap analysis based on 1000 pseudoreplicates. The maximum-likelihood trees were compared using PATRISTIC [47].

We performed Bayesian phylogenetic analyses in BEAST v1.7.5 [48] to estimate the evolutionary rate and timescale of CaMV. The sampling times of the sequences were used as calibrations for the molecular clock. We used Bayes factors to select the best-fitting molecular-clock model and coalescent prior for the tree topology and node times. We compared strict and relaxed (uncorrelated exponential and uncorrelated lognormal) molecular clocks [49] and compared five demographic models (constant population size, expansion growth, exponential growth, logistic growth, and the Bayesian skyline plot). We also tested for clocklike evolution using a regression of root-to-tip distances on viral sampling times in the software Path-O-Gen v1.3 (http://tree.bio.ed.ac.uk/software/pathogen).

Posterior distributions of parameters, including the tree, were estimated by Markov Chain Monte Carlo (MCMC) sampling. Samples were drawn every 10^4^ MCMC steps over a total of 10^8^ steps, with the first 10% of samples discarded as burn-in. Acceptable sampling from the posterior and convergence to the stationary distribution were checked using the diagnostic software Tracer v1.5 (http://tree.bio.ed.ac.uk/software/tracer/).

To estimate substitution rates and divergence times from heterochronous sequence data, the sampling times need to have a sufficient spread in relation to the substitution rate [50]. We investigated the temporal structure in our data sets by comparing our rate estimates with those from ten date-randomized replicates. A data set was considered to have sufficient temporal structure when the mean rate estimate from the original data set was not contained in any of the 95% credibility intervals of the rates estimated from the date-randomized replicates. This follows the approach taken in previous studies [51], [52].

The spatial population dynamics of CaMV through time were inferred in BEAST using a diffusion model with discrete location states [48]. This approach uses an explicit model that describes the migration of CaMV lineages throughout their evolutionary history. The most important pairwise diffusions can be identified using Bayes factors [53]. Using SPREAD [54] and Google Earth (http://www.google.com/earth), we produced a graphical animation of the estimated spatio-temporal movements of CaMV lineages.

### Demographic analyses

DnaSP v5.0 [55] was used to estimate haplotype and nucleotide diversities. Haplotype diversity refers to the frequency and number of haplotypes in the population. Nucleotide diversity estimates the average pairwise differences among sequences. Nonsynomymous (dN) and synonymous (dS) substitution (dN/dS) ratios were calculated for seven ORFs using the Pamilo-Bianchi-Li (PBL) method in MEGA v5 [56]. The program Structure v2.3.4 [57] was used to test for evidence of genetic structure among subpopulations and to identify individuals that were admixed or had migrated among populations. To select the number of clusters that best represented population structure, we performed analyses with 1 to 10 subpopulations (*K* = 1 to 10), sampling from 10^6^ Markov chain steps after a burn-in of 10^5^ steps. We identified the maximum delta-*K* value to determine the best-supported number of subdivisions in the populations [58].

## Results

### Biological characteristics of the CaMV isolates

A total of more than 1000 samples collected during the 2001–2010 growing seasons in Greece, Iran, Japan, and Turkey were tested by DAS-ELISA. About 70 plants of *B. napus* (oilseed rape), *B. oleracea* (cabbage), *B. oleracea* var. *italica* (broccoli), *B. oleracea* var. *botrytis* (cauliflower), *R. sativus* (radish) and other brassicas were found to be infected with CaMV. The viruses were found in commercial fields as well as in home gardens.

*Brassica* and *Raphanus* plants were systemically infected by most isolates. Although they had very minor differences in pathogenicity in *Brassica* and *Raphanus* plants, we concluded that most isolates were of a similar pathotype. In contrast, three (JPNN, JPNS1, and JPNS2) of the ten isolates collected in Japan showed very faint symptoms in both *Brassica* and *Raphanus* plants, and we call these attenuated isolates.

### Genome sequences

The complete genomes of 67 CaMV isolates were sequenced in the present study. The genomes of Eurasian isolates determined in the present study were 7984–8063 nt in length, with ORF lengths of 978–984 nt (ORF I), 459–480 nt (ORF II), 390 nt (ORF III), 1458–1512 nt (ORF IV), 2025–2040 nt (ORF V), 1560–1575 nt (ORF VI), and 285–291 nt (ORF VII). Furthermore, the large intergenic regions located between ORFs VI and VII were 704–784 nt in length, whereas the small intergenic regions located between ORFs V and VI were 103–104 nt in length. All of the motifs reported for different caulimovirus-encoded proteins were found. The new genomic sequences determined in this study are available in DDBJ/EMBL/GenBank databases with accession codes AB863136–AB863202.

### Patristic distance plots

**Figure 1:**
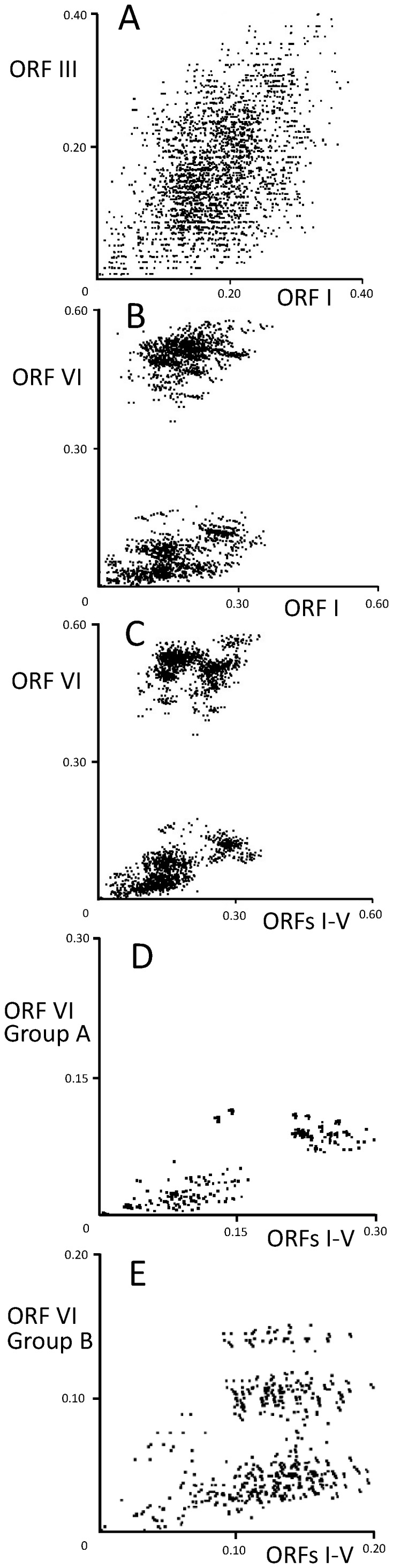
Multidimensional scaling of tree-to-tree patristic distances. ORF I vs ORF III isolates (A); ORF I vs ORF VI isolates (B); ORFs I–V vs ORF VI isolates (C); ORFs I–V Group A vs ORF VI isolates (D); and ORFs I–V Group B vs ORF VI isolates (E).

We made pairwise comparisons of the maximum-likelihood trees of the individual ORFs using PATRISTIC. All pairwise plots of the distances in the trees inferred from the ORFs I, II, III, and IV gave similar patterns. This is illustrated by the plot of ORF I against ORF III distances (Figure 1A), in which the two sets of distances had a linear correlation coefficient of 0.516 (p<0.001). The plots of the ORF V distances against those of ORF I to IV showed that ORF V might have two slightly different but overlapping populations of distances (data not shown). By contrast, plots of the ORF VI distances against those of ORFs I–V, either individually (Figure 1B) or concatenated (Figure 1C), showed that there were two completely distinct lineages of ORF VI, and these were distinct from those in ORF V. Furthermore, plots of the Group A and Group B ORF VI distances against those of the concatenated ORFs I–V (Figure 1D and E) showed that the two sublineages were distinct. The patristic distances of the ORF VII tree gave much more complex patterns when plotted against those of the other ORFs. However, because ORF VII is much shorter than the other ORFs, it is possible that this apparent complexity is an artefact of sampling. Overall, the PATRISTIC plots supported concatenation of ORFs I–V for subsequent evolutionary analyses. We analyzed ORF VI separately and omitted ORF VII from our analyses.

### Recombination analyses

Clear evidence of non-tree like evolution was indicated by the SplitsTree analyses (Figure S1 in File S1). These indicated that there might be recombinant regions in both ORFs I–V and ORF VI. We analyzed the protein-encoding gene sequences of 67 CaMV isolates and nine published sequences for evidence of recombination. Many clear recombination sites were detected throughout the CaMV genomes (Table 1, Figure S2 in File S1). Sites were found at 5′ and 3′ sequences of ORF VI at nt 5996 in the genomes of isolates from Iran and Japan, and at nt 7348 in Greek isolates. Some recombination sites were found in other Turkish genomes, but many were not statistically significant.

**Table 1:** Tentative and clear recombination sites in *Cauliflower mosaic virus* genomes.

Isolate	Position (nt)^a^	ORF	Parental isolate		Recombination detection program^b^	*P*-value^c^
			Major	Minor		
B29	3296-3946	IV–V	TUR50	Unknown (TUR4)	B**S_R_**S_o_P	3.81×10^−9^
	5996-7341	VI	Unknown (TUR50)	TUR4	RGBMC**S_R_**S_o_	2.14×10^−31^
BBC	3259-3946	IV–V	TUR50	Unknown (TUR4)	B**S_R_**S_o_P	6.48×10^−10^
	4214-5995 (UD)	V	Unknown (TUR263)	TUR50	R**G**MCS_o_	3.41×10^−17^
Cabbage S	3298-4078	IV–V	TUR50	Unknown (TUR4)	GB**S_R_**S_o_P	2.02×10^−9^
	6239-74	VI–VII	TUR285	CM1841	RGBMC**S_R_**S_o_	7.43×10^−31^
CM1841	3259-4071	IV–V	TUR50	Unknown (TUR4)	B**S_R_**S_o_P	3.80×10^−10^
	4214-5995	V–VI	Unknown (TUR263)	TUR50	R**G**MC	3.42×10^−15^
CMV-1	3259-4031	IV–V	TUR50	Unknown (TUR4)	**S_R_**S_o_P	2.66×10^−10^
	5887-195	VI–VII	Unknown (TUR4)	TUR50	RGBMC**S_R_**S_o_	7.84×10^−34^
CRO180A	5996-7362	VI	TUR50	Unknown (TUR4)	R**G**BMCS_R_S_o_P	3.10×10^−31^
D/H	5957-82	VI–VII	Unknown (TUR50)	TUR4	RGBMC**S_R_**S_o_P	4.21×10^−35^
GRC83	7240-15	VI–VII	GRC86D	BBC	RG**B**MCS_R_S_o_P	1.32×10^−26^
GRC84B	7240-15	VI–VII	GRC86D	BBC	RG**B**MCS_R_S_o_P	1.37×10^−24^
GRC86B	4318-7239	VI	GRC84B	TUR216	RBM**C**S_R_S_o_P	3.21×10^−10^
GRC86D	7348-615	VI–VII	TUR94	Unknown (CM1841)	RG**B**MCS_R_S_o_	7.28×10^−17^
GRC87E	7348-615	VI–VII	TUR94	Unknown (CM1841)	RG**B**MCS_R_S_o_P	3.18×10^−13^
GRC87G	7348-615	VI–VII	TUR94	Unknown (CM1841)	RG**B**MCS_R_S_o_	4.21×10^−14^
GRC91B	7348-615	VI–VII	TUR94	Unknown (CM1841)	RG**B**MCS_R_S_o_	2.63×10^−15^
GRC92A	7348-615	VI–VII	TUR94	Unknown (CM1841)	RG**B**MCS_R_S_o_	7.99×10^−15^
GRC92C	7348-615	VI–VII	TUR94	Unknown (CM1841)	RG**B**MCS_R_S_o_	1.83×10^−14^
GRC92D	7348-504-	VI–VII	TUR94	Unknown (CM1841)	RG**B**MCS_R_S_o_	1.15×10^−17^
IRN1	5969-102	VI–VII	TUR50	Unknown (TUR4)	RGBMC**S_R_**S_o_	2.07×10^−35^
IRN2	5969-102	VI–VII	TUR50	Unknown (TUR4)	RGBMC**S_R_**S_o_P	7.68×10^−35^
IRN3	5969-102	VI–VII	TUR50	Unknown (TUR4)	RGBMC**S_R_**S_o_	2.07×10^−35^
IRN4	5996-195	VI–VII	TUR50	Unknown (TUR4)	RGBMC**S_R_**S_o_	4.08×10^−34^
IRN5	5996-208	VI–VII	TUR50	Unknown (TUR4)	RGBMC**S_R_**S_o_	1.58×10^−34^
IRN6	5944-180	VI–VII	TUR50	Unknown (TUR4)	RGBMC**S_R_**S_o_	4.70×10^−34^
IRN7	5969-76	VI–VII	TUR50	Unknown (TUR4)	RGBMC**S_R_**S_o_P	8.64×10^−34^
IRN8	5962-208	VI–VII	TUR50	Unknown (TUR4)	RGBMC**S_R_**S_o_	4.48×10^−35^
IRN9	5965-64	VI–VII	TUR50	Unknown (TUR4)	RGBMC**S_R_**S_o_	1.53×10^−33^
IRN10	5967-7342	VI	TUR50	Unknown (TUR4)	RGBMC**S_R_**S_o_	3.77×10^−34^
IRN11	5969-42	VI–VII	TUR50	Unknown (TUR4)	RGBMC**S_R_**S_o_P	1.44×10^−34^
IRN12	5965-7342	VI	TUR50	Unknown (TUR4)	RGBMC**S_R_**S_o_	2.59×10^−34^
IRN13	5965-180	VI–VII	TUR50	Unknown (TUR4)	RGBMC**S_R_**S_o_	1.45×10^−33^
IRN14	5952-99	VI–VII	TUR50	Unknown (TUR4)	RGBMC**S_R_**S_o_	2.46×10^−33^
IRN18	5969-212	VI–VII	TUR50	Unknown (TUR4)	RGBMC**S_R_**S_o_P	1.57×10^−36^
IRN19	5965-64	VI–VII	TUR50	Unknown (TUR4)	RGBMC**S_R_**S_o_	1.18×10^−35^
IRN21	5996-180	VI–VII	TUR50	Unknown (TUR4)	RGBMC**S_R_**S_o_	2.93×10^−34^
JPNHGB340	3259-3946	IV–V	TUR50	Unknown (TUR4)	B**S_R_**S_o_P	5.12×10^−9^
	5996-7341	VI	Unknown (TUR4)	TUR50	RGBMC**S_R_**S_o_	5.81×10^−33^
JPNKWB778	3265-3946	IV–V	TUR50	Unknown (TUR4)	B**S_R_**S_o_P	3.72×10^−9^
	5965-7341	VI	Unknown (TUR4)	TUR50	RGBMC**S_R_**S_o_	3.35×10^−33^
JPNM	4214-5964	V–VI	Unknown (TUR263)	TUR50	R**G**MC	1.26×10^−15^
JPNN	5996-7361	VI	Unknown (TUR4)	TUR50	RGBMC**S_R_**S_o_	1.35×10^−34^
JPNS1	3259-3946	IV–V	TUR50	Unknown (TUR4)	**S_R_**S_o_P	9.43×10^−9^
	5996-269	VI–VII	Unknown (TUR50)	TUR4	RGBMC**S_R_**S_o_	1.19×10^−36^
JPNS2	3259-3946	IV–V	TUR50	Unknown (TUR4)	**S_R_**S_o_P	5.74×10^−9^
	5996-269	VI–VII	Unknown (TUR50)	TUR4	RGBMC**S_R_**S_o_	1.19×10^−36^
JPNUV1	4214-5964	V–VI	Unknown (TUR263)	TUR50	R**G**MC	1.26×10^−15^
JPNUV26	4214-5964	V–VI	Unknown (TUR263)	TUR50	R**G**MC	1.15×10^−16^
JPNTKD762	3242-3989	IV–V	TUR50	Unknown (TUR4)	**S_R_**S_o_P	2.17×10^−8^
	5881-210	VI–VII	Unknown (TUR50)	TUR4	RGBMC**S_R_**S_o_	5.12×10^−35^
NY8153	3296-3946	IV–V	TUR50	Unknown (TUR4)	B**S_R_**S_o_P	3.20×10^−11^
	2104 (UD) - 5896 (UD)	IV–VI	Unknown (TUR263)	TUR50	**S_R_**S_o_P	8.95×10^−13^
	5909-164	VI–VII	Unknown (TUR4)	TUR50	RGBMC**S_R_**S_o_	5.70×10^−35^
TUR2	399-1261	I–II	TUR249	TUR59	RB**S_R_**	2.87×10^−5^
TUR34	4438-5876	V–VI	Unknown (TUR285)	TUR278	**S_R_**S_o_	1.39×10^−6^
TUR59	4511-5948	V–VI	TUR278	Unknown (TUR285)	M**S_R_**S_o_	4.61×10^−7^
	5996-164	VI–VII	TUR4	Unknown (TUR50)	RGBMC**S_R_**P	8.65×10^−34^
TUR214	1772-2108	III–IV	TUR2	TUR12	B**S_R_**S_o_P	2.49×10^−6^
TUR216	2832-4937 (UD)	IV–V	TUR249	Unknown (TUR2)	B**S_R_**S_o_	2.03×10^−16^
	5324-7347	VI	Unknown (TUR306)	GRC92D	**M**	1.90×10^−5^
TUR220	5539-6357	VI	TUR81	Unknown (TUR285)	**R**GB	5.22×10^−5^
TUR239	34 (UD) -1034	I	Unknown (TUR4)	TUR244	RGB**S_R_**S_o_	3.09×10^−10^
	1857 (UD) -2799	V	GRC83	Unknown (IRN2)	**S_R_**S_o_P	3.71×10^−5^
	4365-5326 (UD)	V–VI	TUR50	TUR4	BM**S_R_**S_o_P	6.57×10^−11^
TUR289	471 (UD) -2485	I–IV	TUR84	Unknown (TUR306)	RGBMC**S_R_**S_o_	2.00×10^−9^
TUR306	1831-2512	III–IV	Unknown (TUR94)	TUR84	B**S_R_**S_o_	4.52×10^−9^
W260	3259-3946	IV–V	TUR50	Unknown (TUR4)	B**S_R_**S_o_P	2.54×10^−9^
Xinjing	627-1661	I–III	Unknown (IRN19)	IRN21	R**B**S_o_P	1.96×10^−5^

a Recombination sites detected in the CaMV genomes by the recombination detection programs (listed in column 6), from the aligned sequences of the likely recombinant and its ‘parental isolates’. The nucleotide position shows locations of individual genes numbered as in Xinjing genome (AF140604). UD; Undetermined.

b Recombinant isolates identified by the recombination detection programs: R (RDP), G (GENECONV), B (BOOTSCAN), M (MAXCHI), C (CHIMAERA) and S_R_ (SISCAN) programs in RDP4, and S_O_ (SISCAN total nucleotide site analysis) in original SISCAN version 2 and P (PHYLPRO) programs. The analyses were done using default settings and a Bonferroni-corrected *P*-value cut-off of 0.01 in RDP4.

c The reported *P*-value is for the program in bold type and underlined in RDP4 and is the smallest *P*-value among the isolates calculated for the region in question. *P*-values smaller than 1.0×10^−5^ are listed.

### Phylogenetic analyses

Networks and phylogenetic trees were inferred from concatenated ORFs I–V and from ORF VI. The network inferred from ORFs I–V had short internal branches (Figure S1A in File S1). In contrast, the ORF VI sequences showed two major lineages of CaMV separated by long branches (Figure S1B and Figure S3 in File S1). Each of the subgroups in ORFs I–V and ORF VI contains isolates collected in a geographically confined area.

The major differences between trees from ORFs I–V and from ORF VI are found in the relationships among the subgroups, not the subgroup membership. The maximum-likelihood bootstrapping analysis showed strong support for the various nodes in the ORF VI tree (as in Figure S3 in File S1). In contrast, the tree from ORFs I–V only had strong support at the subgroup level, with the basal nodes having support values below 30%. ORFs I–V and ORF VI yielded maximum-likelihood trees with very different relative branch lengths. In the ORF VI tree, the two basal branches span two-thirds of the mean root-to-tip distance of the tree, compared with only one-tenth in the ORFs I–V tree.

The ORF VI tree partitions most of the sequences into two major groups: Group A consists of Iranian and Japanese/North American/European subgroups, and Group B consists of Greek, Turkish and Iranian subgroups. Although most of the isolates from each country were placed into a single subgroup, those of Iran fell into two. Interestingly, the Iran II isolates that clustered with Turkish isolates in Group B came from the Khorasan Razavi district (see Table S1 in File S1), which is in north-eastern Iran and is not adjacent to Turkey. The topology of Group B showed a geographically hierarchical pattern of evolution, with the Turkish population diverging from the Greek population, and the Iranian population diverging from the Turkish population.

### Genetic population structure

We compared the haplotype and nucleotide diversities of CaMV populations and subpopulations in each country (data not shown). The haplotype diversity in most groups exceeded 0.95. The nucleotide diversity of ORF VI from the Japanese samples in Group A was greater (0.03849) than those of Iran and USA, whereas greater diversity was found in the Greek samples in Group B (although only a small number of Greek isolates were used for these calculations). Nucleotide diversity was highest in Iran (0.06934). In ORFs I–V, nucleotide diversity was higher in Turkey (0.02776) than in Greece, Iran, or Japan. In estimating these genetic differences, we assumed that the population of each country evolved independently, although the sampling area in each country might influence our estimates.

**Figure 2:**
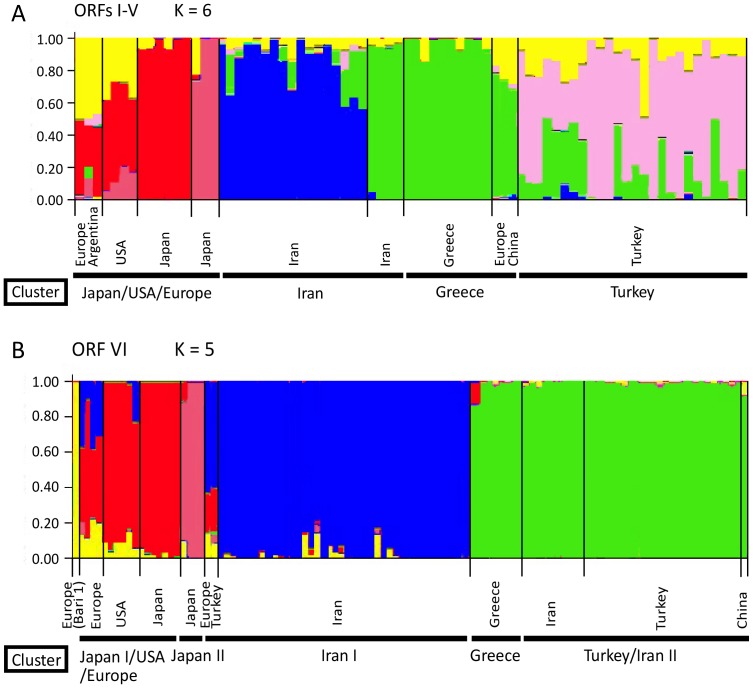
Cluster-based analysis of population subdivision using Structure. The results are grouped by population of origin for each individual. Each individual is represented by a column. The number of clusters is indicated by the value of *K*: ORFs I–V, *K* = 6 (A), ORF VI, *K* = 5 (B). The colour proportion for each bar represents the posterior probability of assignment of each individual to one of six clusters (A) and one of five clusters (B) of genetic similarity. Clusterings correspond to those shown in Figure S1 in File S1.

The cluster-based method implemented in Structure was used to identify individuals that were admixed or had migrated among brassica-infecting CaMV populations. Our analysis supported six subpopulations in ORFs I–V (Figure 2A) and five in ORF VI (Figure 2B). Many individuals contain substantial numbers of nucleotide polymorphisms that are apparently characteristic of ORFs I–V subpopulations, that are colour-coded in Figure 2. The Japan/USA/Europe cluster consisted of yellow, red, and dark pink subpopulations, and the Japanese isolates seemed to be divided into two subpopulations. On the other hand, the Iranian cluster consisted of yellow, green, and blue subpopulations, with the last two being dominant. Turkish clusters consisted of yellow, light pink, and green populations, with the light pink subpopulation being predominant. All of the clusters included the yellow subpopulation, and this might be ancestral the ancestral population. Most individual clusters have a predominant subpopulation in ORF VI (Figure 2B). The major subpopulations of Japan, Iran, and Turkey were red/dark pink, blue, and green, respectively. The Bari 1 isolate was part of the yellow subpopulation, which might be the ancestral isolate of the CaMV subpopulation seen in the Neighbor-Net tree (Figure S1B in File S1). Although the proportion of the yellow subpopulation was small in all clusters, the subpopulation was admixed with other individuals in all clusters. Our results suggest that CaMV became geographically segregated, but with frequent spread between regions.

### Evolutionary rates and timescales

We used a Bayesian phylogenetic method to estimate the evolutionary rates and timescales for the individual genomic regions. Based on the results of our PATRISTIC analyses, we analyzed a concatenated alignment of ORFs I–V and a separate alignment of ORF VI. The best-supported demographic models were exponential growth for ORFs I–V and constant size for ORF VI (Table S2 in File S1). For both data sets, a relaxed-clock model provided a better fit than the strict-clock model (Table 2). To determine whether there was temporal structure in the ORFs I–V and ORF VI data sets, we fitted a linear regression between collection date and the root-to-tip genetic divergence using Path-O-Gen v1.3 (Figure S4 in File S1). For ORFs I–V and ORF VI, we obtained respective *R*-squared values of −0.201 and 0.160, and respective *P*-values of 0.104 and 0.119. These results indicate that the relationship between collection date and sampling time is not significant, so the molecular clock hypothesis is rejected for these data sets.

**Table 2:** Details of the data sets used for estimation of nucleotide substitution rate and time to the most recent common ancestor for *Cauliflower mosaic virus*.

Parameter	Open reading frame	
	I–V	VI
Best-fit substitution model	GTR+I+Γ_4_	GTR+I+Γ_4_
Best-fit molecular clock model	Relaxed Uncorrelated Exponential	Relaxed Uncorrelated Exponential
Best-fit population growth model	Exponential growth	Constant size
Sequence length (nt)	5106	1269
No. of sequences	66	97
Sampling date range	1960–2010	1960–2012
Chain length (in millions)	100	100
TMRCA^a^ (years)	491 (86–1270)	431 (113–886)
Substitution rate (nt/site/year)	1.71×10^−4^ (1.45×10^−5^–3.87×10^−4^)	5.81×10^−4^ (2.47×10^−4^–9.47×10^−4^)
dN/dS^b^	0.069	0.201
No. of variable sites	1074	448

a Time to the most recent common ancestor.

b Nonsynomymous (dN) and synonymous (dS) substitution (dN/dS) ratios were calculated for seven ORFs using the Pamilo-Bianchi-Li (PBL) method in MEGA v5 [56].

**Figure 3:**
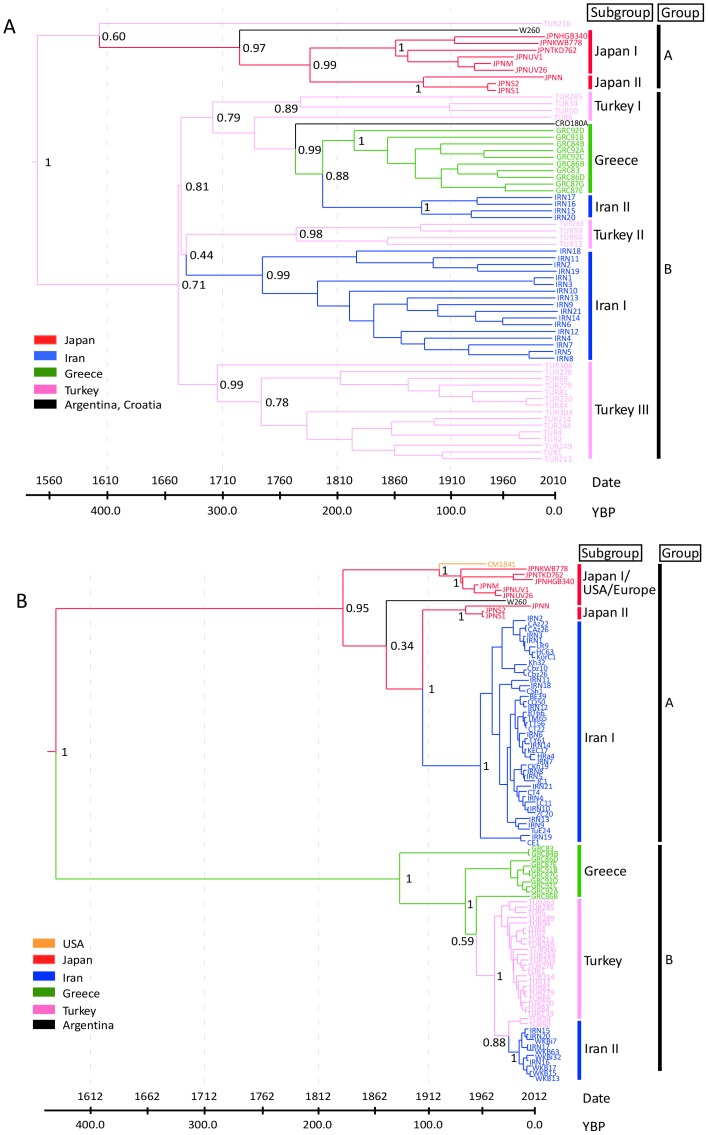
Bayesian phylogenetic estimates from ORFs I–V and ORF VI of *Cauliflower mosaic virus*. Maximum-clade–credibility trees from BEAST analyses of 66 and 97 isolates of ORFs I–V (A) and ORF VI (B), respectively. Branch colours correspond to the most probable geographic location of their descendent nodes.

Nonetheless our analyses of date-randomized replicates revealed that the sampling times of ORFs I–V and ORF VI had sufficient temporal structure for calibration of the molecular clock (Figure S5 in File S1). This was indicated by the smaller 95% credibility intervals of the rate estimates from the original data set compared with the date-randomized replicates. In addition, the mean posterior rate estimates from the original data were not contained with the 95% credibility intervals of the rate estimates from the date-randomized replicates. The mean estimated substitution rates were 1.71×10^−4^ subs/site/year for ORFs I–V and 5.81×10^−4^ subs/site/year for ORF VI (Table 2). Estimates of the age of the root were 491 years for ORFs I–V and 431 years for ORF VI (Table 2, Figure 3).

### Patterns of viral migration

**Figure 4:**
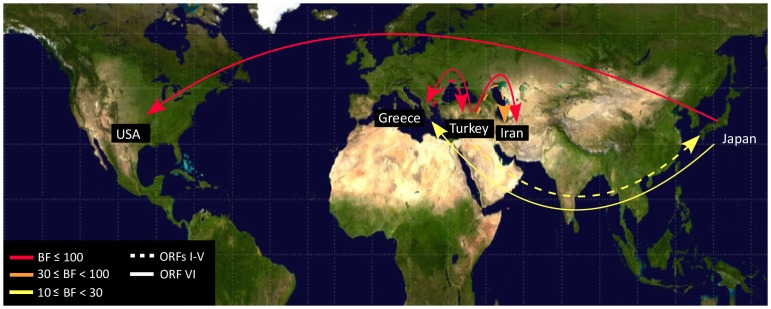
Patterns of *Cauliflower mosaic virus* migration jointly estimated across the two ORF regions. ORFs I–V and ORF VI migrations are shown by solid and dashed lines. Lines connecting discrete regions indicate statistically supported ancestral state changes and their thicknesses denote statistical support. There are five categories of support. In increasing order, line thicknesses indicate 6≤BF<10 (positive support); 10≤BF<30 (strong support); 30≤BF<100 (very strong support); and BF≥100 (decisive support). Migration line was not shown when they were represented by only a single sample.

Our Bayesian phylogenetic analysis of the origin and global spread of CaMV showed strong Bayes factor (BF) support from ORFs I–V hat the virus had spread from Turkey to Greece (BF = 205) and to Iran (BF = 61) (Figure 4). There was also some support for spread from Turkey to Japan (BF = 14). The ORF VI data supported spread from Greece to Turkey (BF = 230) and to Iran (BF = 128), and from Japan to USA (BF = 112).

## Discussion

We aimed to understand the migration dynamics and spread of CaMV in their natural hosts by utilizing over 50 years of surveillance data. Our analyses show that the samples from Europe, Japan, Middle East and USA, including the regions where various Brassicaceae were first domesticated, seems to have captured a significant sample of the global genetic diversity of CaMV. The presence of as-yet-uncollected CaMV infecting different non-brassica plant species may have biased our analysis against the detection of heterotopic processes. We recently presented a similar case study for TuMV evolution using wild orchid and brassica isolates [9].

Our comparisons of the ML trees of the individual ORFs using PATRISTIC showed that the ORFs I–V shared similar evolutionary histories, and this was different from that of ORF VI (Figure 1). The ORF I–V proteins are expressed from 35S RNA, whereas ORF VI protein is from 19S RNA. ORF VI protein is the major component of cytoplasmic inclusion bodies and the structures called viroplasms, which are thought to be ‘virion factories’. Additionally, this protein is an essential determinant of host range, affects symptom severity [20], and is known to transactivate the translation of ORFs I–V from the polycistronic 35S protein [20], [59]. Interestingly, attenuated isolates of three Japanese JPNN, JPNS1, and JPNS2 were found in the present study, and the isolates grouped together in the ORF VI tree (Figure S1B in File S1).

Recombination is an important source of genetic variation not only for CaMV [30], [60] but also for many other plant viruses [3], [13], [61]–[63]. We report several phylogenetic patterns that might have resulted from recombination in CaMV and that have not previously been found in the isolates from North America [30]. Additionally, although recombination sites have not been found in the ORF VI region [64], we found that many isolates from Europe, Iran, Japan and USA isolates were recombinants, with sites located at the 5′ and 3′ ends of ORF VI (Table 1, Figure S2 in File S1). Our results suggest that these two sites are recombination hot spots in CaMV. The recombination hot spot at the 5′ end in ORF VI is located in the middle of reported virulence/avirulence [65] and pathogenicity domains [59], [66]. The present geographical distributions of the various CaMV recombinant lineages imply that there have been complex patterns of CaMV movement throughout the world.

Our estimates of the genetic population structure have shown that there has been frequent spread between regions (Figure 2). However, the structure of ORF VI (Figure 2B) showed clear geographical segregation at the primary divergence of the CaMV population, which was not shown by ORFs I–V (Figure 2A). The same divergences were shown by the Neighbor-Net trees of the same data (Figure S1 in File S1). Our Bayesian phylogenetic analysis revealed that ORFs I–V and ORF VI support different local migration patterns for CaMV. For instance, ORFs I–V showed that CaMV migrated from Turkey to Greece and Iran, whereas ORF VI data set showed that the virus from Greece and then spread to Turkey or Iran. This suggests that there was insufficient phylogenetic signal to reveal unequivocally the complex patterns of migration in the CaMV populations in the past. The Neighbour-Net tree (Figure S1B in File S1) was estimated from ORF VI sequences that included one from the Italian Bari1 isolate. The position of this isolate in the ORF VI tree suggests that there might be a third distinct CaMV population that is yet to be sampled and sequenced. The different migration patterns in different regions might reflect characteristics of CaMV transmission and geographical barriers. CaMV is transmitted by aphids in a semi-persistent manner and they are able to only carry the virus for a short time. Mountains, deserts, country-dependent agriculture crops and growing conditions of crops may present obstacles to the spread of aphids, thus limiting the spread of the virus. Physical obstacles have also been reported to be responsible for the strain localization of *Rice yellow mottle virus* [67] and *Tobacco vein banding mosaic virus* [68].

CaMV mainly infects brassica crops, including cabbage, broccoli and cauliflower. Non-heading cabbages and kale were probably domesticated before 1000 BC in Eurasia [69], but were not taken to North America and Japan until the 17th and 19th centuries respectively. Broccoli and perhaps cauliflower originated from kale, and first appeared in the east Mediterranean. Broccoli and cauliflower spread from Italy to other European countries around the 16th to 19th centuries, prior to their introduction into North America and Japan in 19th to 20th centuries [70], [71]. Our estimate of the divergences in the tree of ORF VI shows that the primary divergence was around 450 years ago, but the divergences of the subgroup lineages occurred about 100–200 years ago (Figure 3B). Thus our well-supported estimate of the time to the most recent common ancestor of CaMV lineages based on the ORFs VI sequences is consistent with the global trade in broccoli, cauliflower and other brassica species grown as antiscorbutics, from Europe to other parts of the world. This timing also suggests that aphids were not responsible for the primary global spread of CaMV. Further global sampling of CaMV isolates is needed to confirm these results and the discrepancy between the topologies of the ORF I–V and ORF VI trees, nonetheless the age of the ancestor of CaMV fits neatly with the timescale of migration of brassica crops across the world.

We have interpreted our results while assuming that CaMV has evolved in a straightforward manner. We have concluded that the apparent difference in phylogeny between the ORFs I–V and ORF VI genes results from an inadequate phylogenetic signal in ORFs I–V, as shown by the lack of bootstrap support for the basal nodes of trees estimated from those sequences. However it is important to note that the evolution of CaMV, a pararetrovirus, may be unusual. CaMV has an unusually high recombination rate [60], and its populations have very large effective sizes [72]. Another paraeretrovirus, *Banana streak virus*, exists as both a virus and as endogenous elements integrated within the host genome with, probably, completely different evolutionary rates [73]. It is also noteworthy that the 35S promoter that is widely used in transgenic plant research includes much of ORF VI [74]. Thus, the unexpected should be expected in studies of the molecular phylogenetics of caulimovirids, not only in the gene sequences themselves but also in their behavior in the methods used to analyze them.

In conclusion, our study has shown that (i) recombination is common in CaMV; (ii) ORFs I–V and ORF VI of its genome show different evolutionary patterns; (iii) the ORFs are evolving at a rate in the range of 1.71–5.81×10^−4^ substitutions/site/year, which is similar to that of RNA and ssDNA viruses; (iv) ORF VI is the most rapidly evolving ORF; (v) there is evidence of at least four geographically confined lineages of CaMV; (vi) CaMV probably spread from a single population to other parts of the world around 400–500 years ago; (vii) CaMV is widely distributed in Eurasian countries; and (viii) there is evidence of frequent spread between Turkey and neighboring countries, and similarly between Japan and the USA. This is the first report on the spatial and temporal spread of a plant pararetrovirus.

## Supporting Information

File S1 **Figures S1–S5 & Tables S1–S2.**
**Figure S1.** Phylogenetic evidence for recombination among *Cauliflower mosaic virus* from the Europe, Japan, Middle East (Iran and Turkey) and USA. ORFs I–V (A) and ORF VI (B). Neighbor-Net network analysis was performed using SplitsTree4. *Horseradish latent virus* is used as the outgroup. Formation of a reticular network rather than a single bifurcated tree is suggestive of recombination. The isolates obtained in this study are listed in Table S1 in File S1.
**Figure S2.** Recombination analysis by RAT plot. Each blue line represents a pairwise sequence comparison. The red curve represents the estimated proportion of recombinants at each position in the alignment. The red vertical lines denote estimated positions of recombination breakpoints, which approximately match the boundaries of the ORF VI region. The estimated nucleotide positions of the recombination sites are shown relative to the 5′ end of the genome, using numbering of the gapped aligned sequences with gaps removed (see Materials and methods). Recombination sites in parentheses are shown relative to the 5′ end of the genome using numbering of the sequence of the Xinjing isolate.
**Figure S3.** Maximum-likelihood tree estimated from ORF VI of 105 non-recombinant *Cauliflower mosaic virus* isolates. Nodes are labelled with bootstrap support percentages.
**Figure S4.** Regression of root-to-tip distance (inferred from Maximum-likelihood trees) against year of isolation for the gene with the smallest number of sequences in each ORF region.
**Figure S5.** Estimates of nucleotide substitution rates. Mean estimates and 95% credibility intervals are shown. These were estimated from 66 ORFs I–V and 97 ORF VI (see text). In each set of estimates, the first is based on the original data, whereas the remaining ten values are from date-randomized replicates. The 95% credibility intervals of the estimates from the date-randomized replicates do not overlap with the mean posterior estimate from the original data set. In addition, the lower tails of the credibility intervals are long and tend towards zero. These features suggest that there is sufficient temporal structure in the original data sets for rate estimation.
**Table S1.** *Cauliflower mosaic virus* isolates analyzed in this study.
**Table S2.** Detailed results from BEAST analyses of *Cauliflower mosaic virus*.
(PDF)
