# Correction: The Temporal Evolution and Global Spread of *Cauliflower mosaic virus*, a Plant Pararetrovirus

**DOI:** 10.1371/journal.pone.0095410

**Published:** 2014-04-10

**Authors:** The PLOS ONE Staff

Figure 4 is incorrect. The authors have provided a corrected version here.

**Figure 4:**
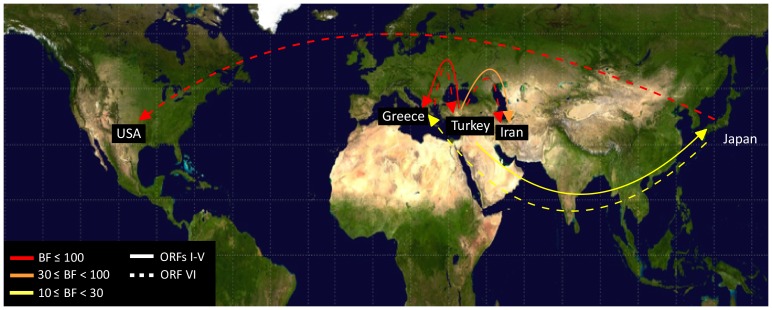
Patterns of *Cauliflower mosaic* virus migration jointly estimated across the two ORF regions. ORFs I–V and ORF VI migrations are shown by solid and dashed lines. Lines connecting discrete regions indicate statistically supported ancestral state changes and their thicknesses denote statistical support. There are five categories of support. In increasing order, line thicknesses indicate 6≤BF<10 (positive support); 10≤BF<30 (strong support); 30≤BF<100 (very strong support); and BF≥100 (decisive support). Migration line was not shown when they were represented by only a single sample.

There is a typo in the third sentence of the “Patterns of viral migration” subsection of the Results section. The sentence currently reads:

“The ORF VI data supported spread from Greece to Turkey (BF  =  230) and to Iran (BF  =  128), and from Japan to USA (BF  =  112).”

The correct sentence should read:

“There was also some support for spread from Turkey to Japan (BF  =  14). The ORF VI data supported spread from Greece to Turkey (BF =  230) and Turkey to Iran (BF =  128), and from Japan to USA (BF  =  112).”
