# Supplementary material for: The Temporal Evolution and Global Spread of Cauliflower mosaic virus, a Plant Pararetrovirus
Source: PLoS One. 2014 Jan 21;9(1):e85641. doi: 10.1371/journal.pone.0085641 (PMC3897471; doi:10.1371/journal.pone.0085641)
Supplement: File S1 — Figures S1–S5 & Tables S1–S2. Figure S1. Phylogenetic evidence for recombination among Cauliflower mosaic virus from the Europe, Japan, Middle East (Iran and Turkey) and USA. ORFs I–V (A) and ORF VI (B). Neighbor-Net network analysis was performed using SplitsTree4. Horseradish latent virus is used as the outgroup. Formation of a reticular network rather than a single bifurcated tree is suggestive of recombination. The isolates obtained in this study are listed in Table S1 in File S1. Figure S2. Recombination analysis by RAT plot. Each blue line represents a pairwise sequence comparison. The red curve represents the estimated proportion of recombinants at each position in the alignment. The red vertical lines denote estimated positions of recombination breakpoints, which approximately match the boundaries of the ORF VI region. The estimated nucleotide positions of the recombination sites are shown relative to the 5′ end of the genome, using numbering of the gapped aligned sequences with gaps removed (see Materials and methods). Recombination sites in parentheses are shown relative to the 5′ end of the genome using numbering of the sequence of the Xinjing isolate. Figure S3. Maximum-likelihood tree estimated from ORF VI of 105 non-recombinant Cauliflower mosaic virus isolates. Nodes are labelled with bootstrap support percentages. Figure S4. Regression of root-to-tip distance (inferred from Maximum-likelihood trees) against year of isolation for the gene with the smallest number of sequences in each ORF region. Figure S5. Estimates of nucleotide substitution rates. Mean estimates and 95% credibility intervals are shown. These were estimated from 66 ORFs I–V and 97 ORF VI (see text). In each set of estimates, the first is based on the original data, whereas the remaining ten values are from date-randomized replicates. The 95% credibility intervals of the estimates from the date-randomized replicates do not overlap with the mean posterior estimate from the original dat [file pone.0085641.s001.pdf]

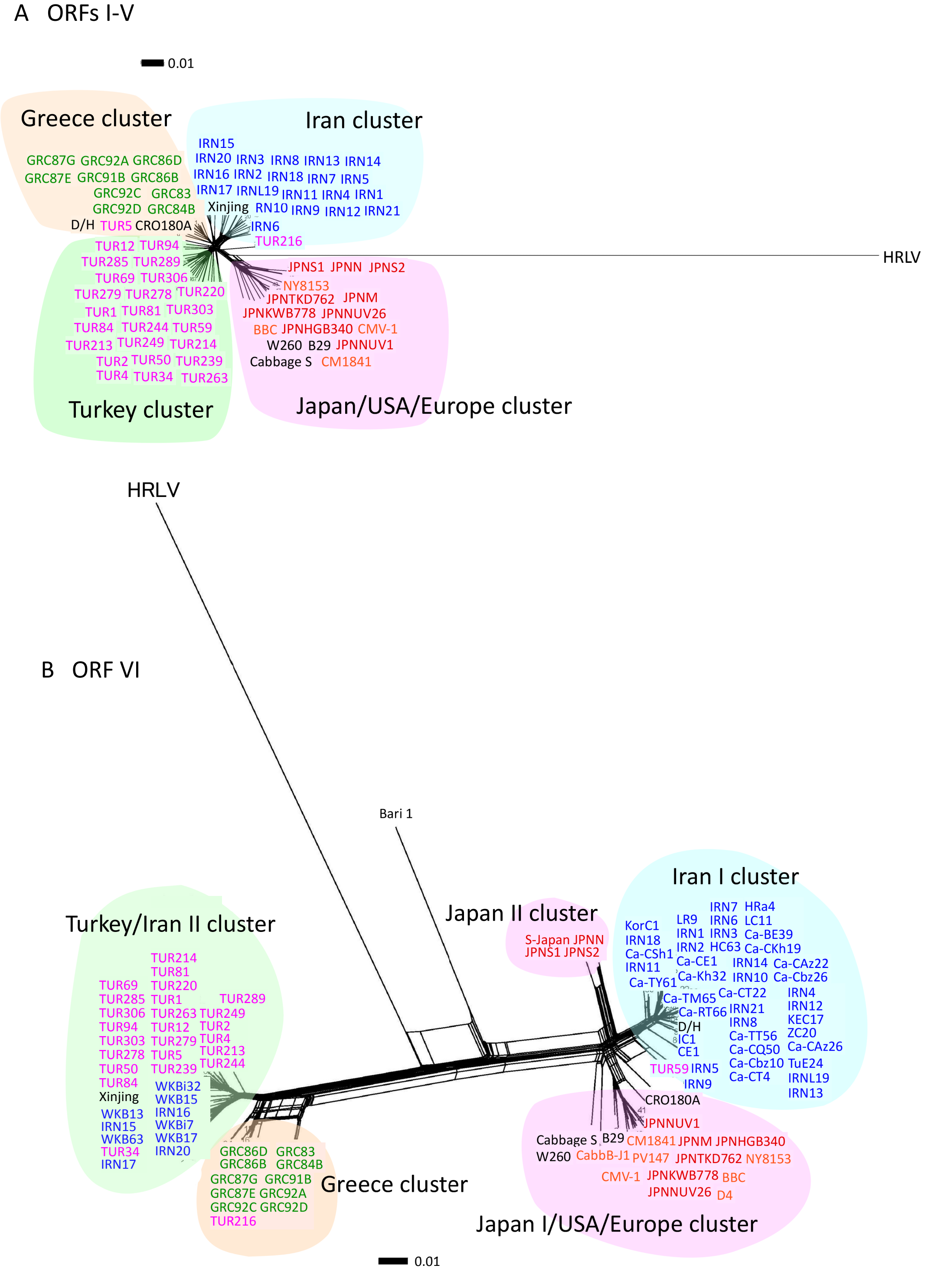
A ORFs I-V
0.01
Greece cluster
Iran cluster
Turkey cluster
Japan/USA/Europe cluster
HRLV
GRC87G GRC92A GRC86D
GRC87E GRC91B GRC86B
GRC92C GRC83
GRC92D GRC84B
D/H TUR5 CRO180A
IRN15
IRN20 IRN3 IRN8 IRN13 IRN14
IRN16 IRN2 IRN18 IRN7 IRN5
IRN17 IRNL19 IRN11 IRN4 IRN1
Xinjing RN10 IRN9 IRN12 IRN21
IRN6
TUR216
TUR12 TUR94
TUR285 TUR289
TUR69 TUR306
TUR279 TUR278 TUR220
TUR1 TUR81 TUR303
TUR84 TUR244 TUR59
TUR213 TUR249 TUR214
TUR2 TUR50 TUR239
TUR4 TUR34 TUR263
JPNS1 JPNN JPNS2
NY8153
JPNTKD762 JPNM
JPNKWB778 JPNNUV26
BBC JPNHGB340 CMV-1
W260 B29 JPNNUV1
Cabbage S CM1841
B ORF VI
HRLV
Bari 1
Turkey/Iran II cluster
Japan II cluster
Iran I cluster
Greece cluster
Japan I/USA/Europe cluster
TUR214
TUR81
TUR69 TUR220
TUR285 TUR1 TUR289
TUR306 TUR263 TUR249
TUR94 TUR12 TUR2
TUR303 TUR279 TUR4
TUR278 TUR5 TUR213
TUR50 TUR239 TUR244
TUR84 WKBi32
Xinjing WKB15
WKB13 IRN16
IRN15 WKBi7
WKB63 WKB17
TUR34 IRN20
IRN17
GRC86D GRC83
GRC86B GRC84B
GRC87G GRC91B
GRC87E GRC92A
GRC92C GRC92D
TUR216
S-Japan JPNN
JPNS1 JPNS2
KorC1 LR9 IRN7 HRa4
IRN18 IRN6 LC11
IRN1 IRN3 Ca-BE39
Ca-CSh1 IRN2 HC63 Ca-CKh19
IRN11 Ca-CE1 IRN14 Ca-CAz22
Ca-TY61 Ca-Kh32 IRN10 Ca-Cbz26
Ca-TM65 Ca-CT22 IRN4
Ca-RT66 IRN21 IRN12
D/H IRN8 KEC17
IC1 Ca-TT56 ZC20
CE1 Ca-CQ50 Ca-CAz26
TUR59 IRN5 Ca-Cbz10 TuE24
IRN9 Ca-CT4 IRNL19
IRN13
CRO180A
JPNNUV1
Cabbage S B29 CM1841 JPNM JPNHGB340
W260 CabbB-J1 PV147 JPNTKD762 NY8153
CMV-1 JPNKWB778 BBC
JPNNUV26 D4
0.01

Figure S1

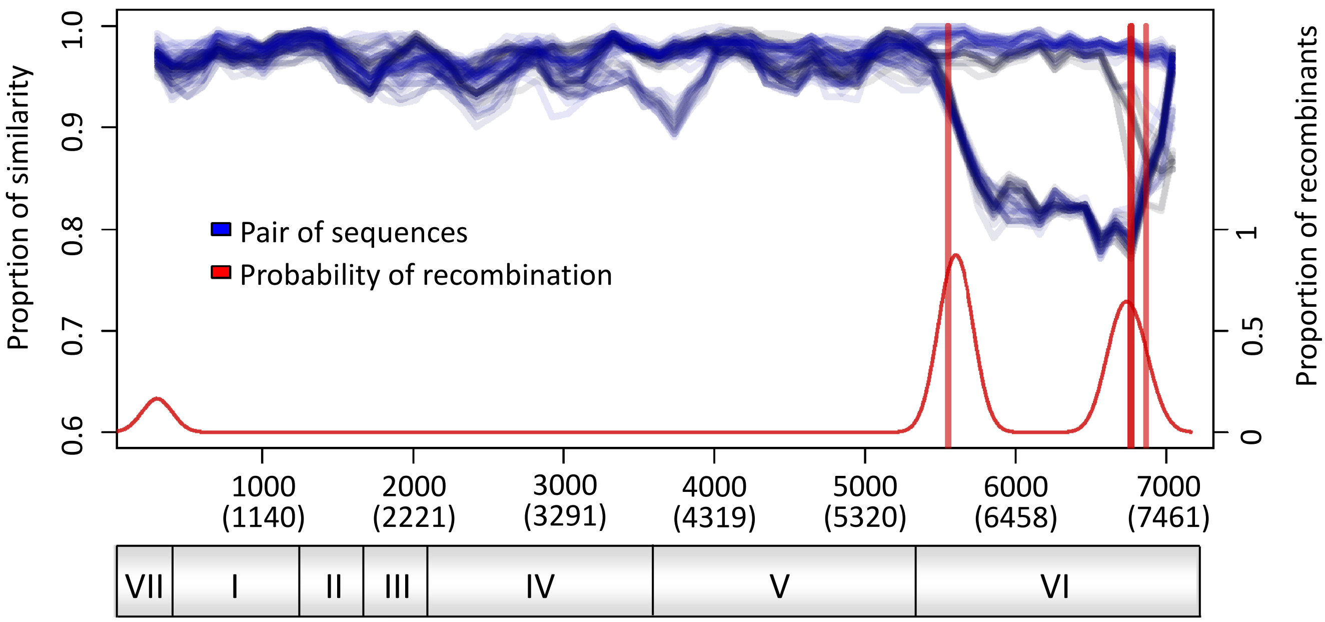
Proprtion of similarity
Proportion of recombinants
1.0
0.9
0.8
0.7
0.6
1
0.5
0
Pair of sequences
Probability of recombination
1000
(1140)
2000
(2221)
3000
(3291)
4000
(4319)
5000
(5320)
6000
(6458)
7000
(7461)
VII
I
II
III
IV
V
VI

Figure S2

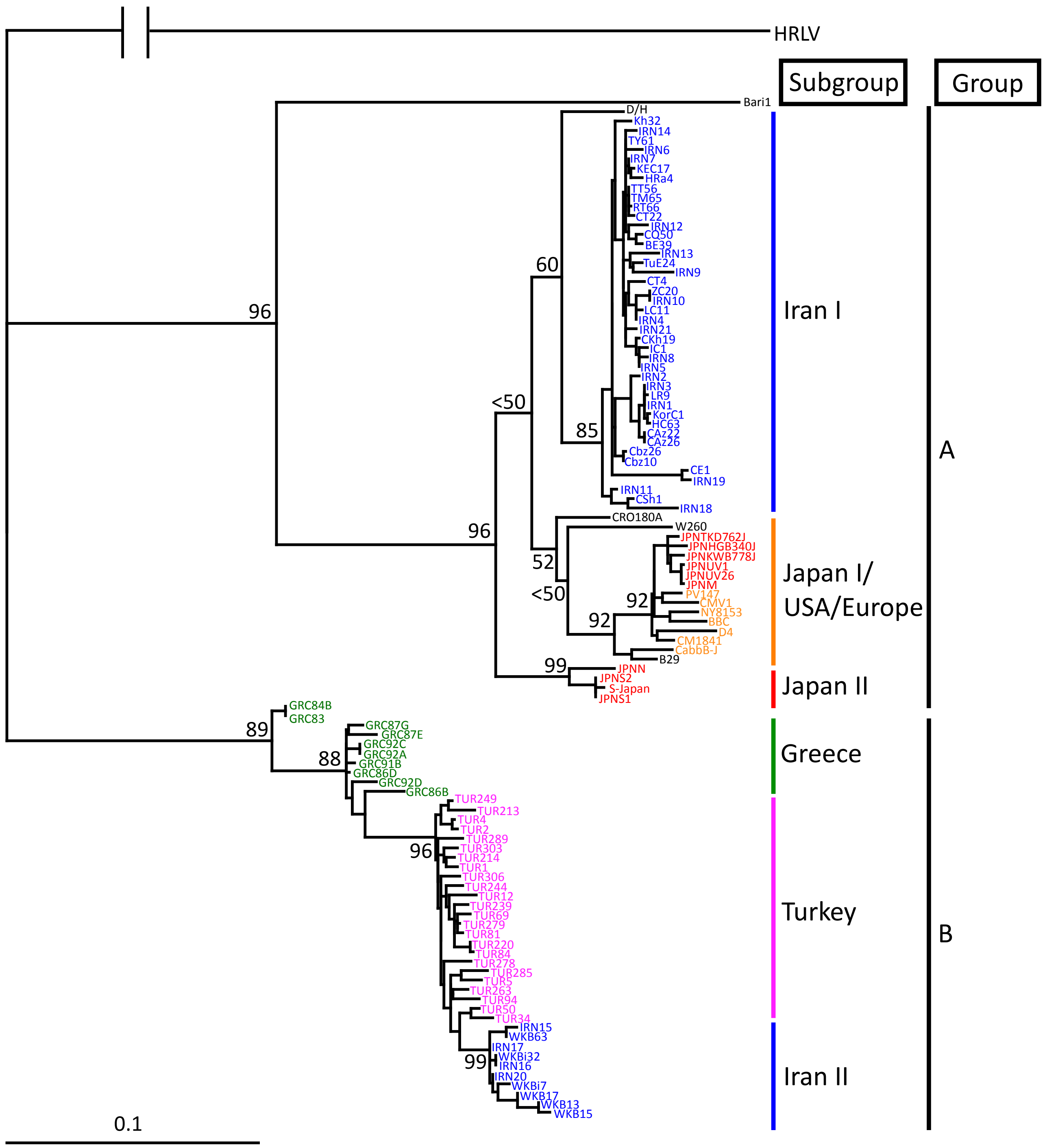
HRLV
Subgroup
Group
Bari1
D/H
Iran I
Japan I/
USA/Europe
Japan II
Greece
Turkey
Iran II
A
B
96
60
<50
85
96
52
<50
92
92
99
89
88
96
99
0.1

Figure S3

A ORFs I-V

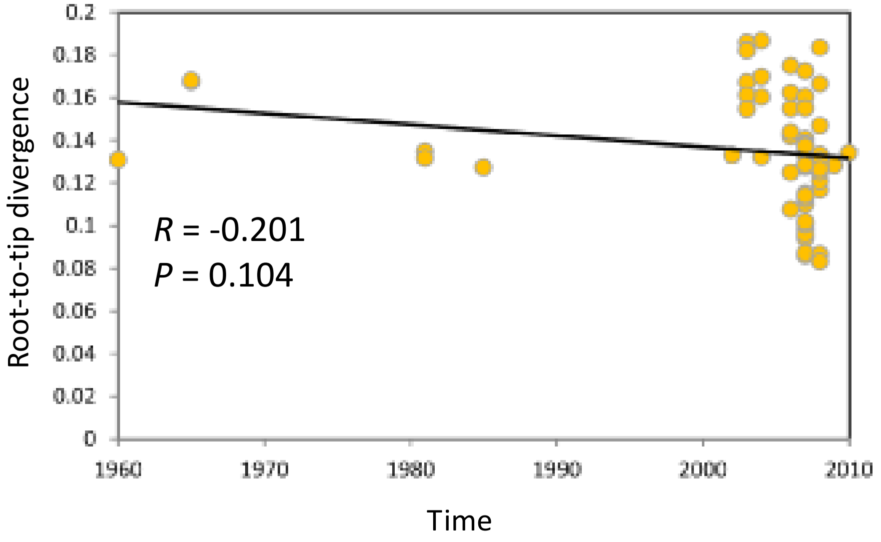

B ORF VI

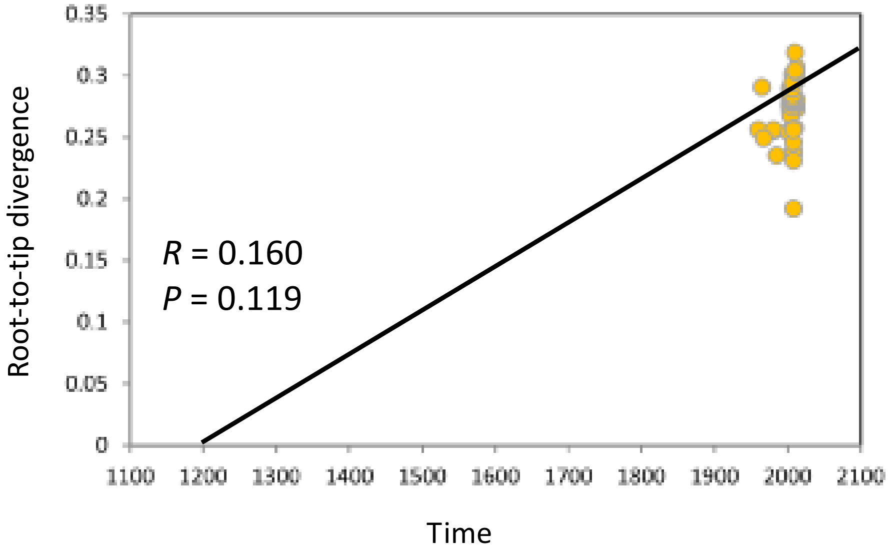

Figure S4

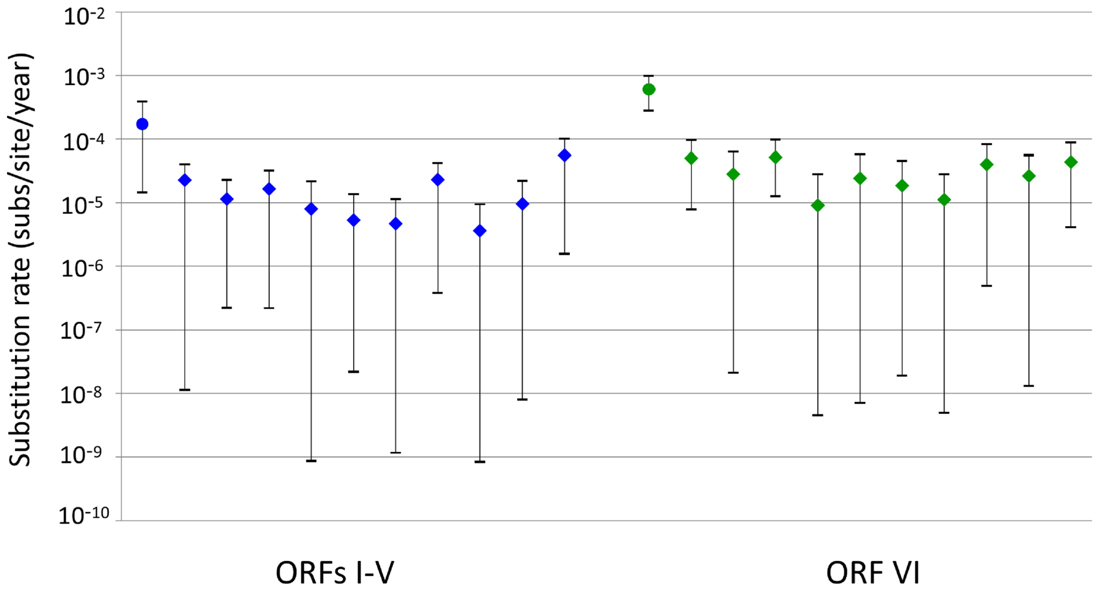

Substitution rate (subs/site/year)
10^{-2}
10^{-3}
10^{-4}
10^{-5}
10^{-6}
10^{-7}
10^{-8}
10^{-9}
10^{-10}
ORFs I-V
ORF VI

Figure S5

Table S1. *Cauliflower mosaic virus* isolates analyzed in this study.

Isolate	Original host	Common name	Location (city, district)	Year of collection	Reference	Sequenced region or Accession no.
Asia						
China						
Xinjing	*Brassica oleracea*	Cabbage	-, Xinjiang	1963	[1], [2]	AF140604 (Full)
Iran						
Ca-BE39	*B. oleracea* var. *italica*	Broccoli	-, Esfahan	2004		DQ870913 (partial ORF VI)
Ca-CAz22	*B. oleracea* var. *botrytis*	Cauliflower	Orumiyeh, Azarbayjan-e-Gharbi	2004	[3]	DQ870907 (partial ORF VI)
Ca-CAz26	*B. oleracea* var. *botrytis*	Cauliflower	Orumiyeh, Azarbayjan-e-Gharbi	2004	[3]	DQ870908 (partial ORF VI)
Ca-Cbz10	*B. oleracea* var. *capitata*	Cabbage	-, Zanjan	2004		DQ870909 (partial ORF VI)
Ca-Cbz26	*B. oleracea* var. *capitata*	Cabbage	-, Zanjan	2004		DQ870911 (partial ORF VI)
Ca-CE1	*B. oleracea* var. *botrytis*	Cauliflower	-, Esfahan	2003	[3]	DQ119041 (partial ORF VI)
Ca-CKh19	*B. oleracea* var. *botrytis*	Cauliflower	-, Khorasan	2004	[3]	DQ870910 (partial ORF VI)
Ca-CSh1	*B. oleracea* var. *botrytis*	Cauliflower	Shiraz, Fars	2003	[3]	DQ119040 (partial ORF VI)
Ca-CQ50	*B. oleracea* var. *botrytis*	Cauliflower	-, Qazvin	2004	[3]	DQ870914 (partial ORF VI)
Ca-CT4	*B. oleracea* var. *botrytis*	Cauliflower	Varamin, Tehran	2004	[3]	DQ870912 (partial ORF VI)
Ca-CT22	*B. oleracea* var. *botrytis*	Cauliflower	Karaj, Tehran	2004	[3]	DQ870915 (partial ORF VI)
Ca-Kh32	*B. oleracea* var. *botrytis*	Cauliflower	-, Khorasan	2004	[3]	EF503597 (partial ORF VI)
Ca-RT66	*R. sativus*	Radish	Karaj, Tehran	2004		DQ870918 (partial ORF VI)
Ca-TM65	*B. rapa*	Turnip	Mahallat, Markazi	2005		DQ870917 (partial ORF VI)
Ca-TT56	*B. rapa*	Turnip	Karaj, Tehran	2004		DQ870919 (partial ORF VI)
Ca-TY61	*B. rapa*	Turnip	Meybod, Yazd	2004		DQ870916 (partial ORF VI)
HC63	*B. napus*	Oilseed rape	Razan, Hamedan	2012	[4]	KF015282 (ORF VI)
HRa4	*R. rogusum*	Annual bastard cabbage	Hamedan, Hamedan	2012	[4]	KF015283 (ORF VI)
IC1	*B. napus*	Oilseed rape	Ilam, Ilam	2012	[4]	KF015284 (ORF VI)
IRN1	*B. oleracea* var. *botrytis*	Cauliflower	Uromia, Azarbayjan-e-Gharbi	2003	this study	AB863136 (Full)
IRN2	*B. oleracea* var. *botrytis*	Cauliflower	Uromia, Azarbayjan-e-Gharbi	2003	this study	AB863137 (Full)
IRN3	*B. oleracea* var. *botrytis*	Cauliflower	-, Qazvin	2003	this study	AB863138 (Full)
IRN4	*B. oleracea* var. *botrytis*	Cauliflower	Varamin, Tehran	2003	this study	AB863139 (Full)
IRN5	*B. pekinensis*	Chinese cabbage	Karaj, Tehran	2003	this study	AB863140 (Full)
IRN6	*B. oleracea* var. *italica*	Broccoli	Falavarjan, Esfahan	2003	this study	AB863141 (Full)
IRN7	*B. oleracea* var. *botrytis*	Cauliflower	Karaj, Tehran	2004	this study	AB863142 (Full)
IRN8	*Matthiola* sp.	Stock	Mahallt, Markazi	2004	this study	AB863143 (Full)
IRN9	*B. rapa*	Turnip	Mahallat, Markazi	2004	this study	AB863144 (Full)
IRN10	*Raphanus rugosum*	Annual bastard cabbage	Vavan, Tehran	2006	this study	AB863145 (Full)
IRN11	*B. napus*	Oilseed rape	Shiraz, Fars	2006	this study	AB863146 (Full)
IRN12	*R. rogusum*	Annual bastard cabbage	Vavan, Tehran	2006	this study	AB863147 (Full)
IRN13	*R. sativus*	Radish	-, Yazd	2006	this study	AB863148 (Full)
IRN14	*B. oleracea* var. *botrytis*	Cauliflower	Falavarjan, Esfahan	2007	this study	AB863149 (Full)
IRN15	*Lepidium sativum*	Garden cress	Mashhad, Khorasan Razavi	2007	this study	AB863150 (Full)
IRN16	*R. sativus*	Radish	Mashhad, Khorasan Razavi	2007	this study	AB863151 (Full)
IRN17	*R. sativus*	Radish	Mashhad, Khorasan Razavi	2007	this study	AB863152 (Full)
IRN18	*R. sativus*	Radish	Shiraz, Fars	2007	this study	AB863153 (Full)
IRN19	*B. oleracea* var. *gongylodes*	Kohlrabi	Shiraz, Fars	2007	this study	AB863154 (Full)
IRN20	*B. rapa*	Turnip	Mashad, Khorasan Razavi	2007	this study	AB863155 (Full)
IRN21	*R. sativus*	Radish	-, Esfahan	2008	this study	AB863156 (Full)
IRNCaCOr1	*B. oleracea* var. *botrytis*	Cauliflower	Uromia, Azarbayjan-e-Gharbi	2004		DQ119036 (ORF II)
IRNCaCQ1	*B. oleracea* var. *botrytis*	Cauliflower	Boin-zahra, Qazvin	2001		DQ119038 (ORF II)
IRNCaCSh1	*B. oleracea* var. *botrytis*	Cauliflower	Shiraz, Fars	2003		DQ119039 (ORF II)
IRNCaME1	*B. oleracea* var. *italica*	Broccoli	-, Esfahan	2003		AY703456 (ORF II)
IRNCaRE4	*R. sativus*	Radish	-, Esfahan	2003		DQ119037 (ORF II)
KEC17	*B. napus*	Oilseed rape	Kermanshah, Kermanshah	2012	[4]	KF015285 (ORF VI)
KOrC1	*B. napus*	Oilseed rape	-, Kordestan	2012	[4]	KF015287 (ORF VI)
LC11	*B. napus*	Oilseed rape	Koram abad, Lorestan	2012	[4]	KF015288 (ORF VI)
LR9	*R. sativus*	Radish	Koram abad, Lorestan	2012	[4]	KF015286 (ORF VI)
TuE24	*B. rapa*	Turnip	-, Esfahan	2012	[4]	KF015289 (ORF VI)
WKBi7	*B. oleracea* var. *capitata*	White cabbage	Birjand, Khorasan-e-Jonubi	2012	[4]	KF015294 (ORF VI)
WKBi32	*B. oleracea* var. *capitata*	White cabbage	Birjand, Khorasan-e-Jonubi	2012	[4]	KF015295 (ORF VI)
WKB13	*B. oleracea* var. *capitata*	White cabbage	Bohnurd, Khorasan-e-Shomali	2012	[4]	KF015290 (ORF VI)
WKB15	*B. oleracea* var. *capitata*	White cabbage	Bohnurd, Khorasan-e-Shomali	2010	[4]	KF015291 (ORF VI)
WKB17	*B. oleracea* var. *capitata*	White cabbage	Bohnurd, Khorasan-e-Shomali	2010	[4]	KF015292 (ORF VI)
WKB63	*B. oleracea* var. *capitata*	White cabbage	Bohnurd, Khorasan-e-Shomali	2012	[4]	KF015293 (ORF VI)
ZC20	*B. napus*	Oilseed rape	-, Zanjan	2012	[4]	KF015296 (ORF VI)
Japan						
JPNHGB340	*B. oleracea*	Cabbage	-, Hyogo	2010	this study	AB863157 (Full)
JPNKWB778	*B. oleracea* var. *italica*	Broccoli	Takamatsu, Kagawa	2004	this study	AB863158 (Full)
JPNM	*B. oleracea*	Cabbage	-, Tokyo	1960	this study	AB863159 (Full)
JPNN	*B. oleracea*	Cabbage	Sendai, Miyagi	2008	this study	AB863160 (Full)
JPNS1	*Armoracia rusticana*	Horseradish	-, Nagano	1965	this study	AB863161 (Full)
JPNS2	*A. rusticana*	Horseradish	-, Nagano	1965	this study	AB863162 (Full)
JPNTKD762	*R. sativus*	Radish	Miyoshi, Tokushima	2002	this study	AB863163 (Full)
JPNUV1	*B. oleracea*	Cabbage	-, Ibaraki	1981	this study	AB863164 (Full)
JPNUV26	*B. oleracea*	Cabbage	-, Ibaraki	1981	this study	AB863165 (Full)
S-Japan	*A. rusticana*	Horseradish	Yokohama, Kanagawa	Not known	[5]	X14911 (ORF VI)

[1] Xie *et al*., 1979, [2] Fang *et al*., 1985, [3] Farzadfar *et al*., 2007, [4] Farzadfar and Pourrahim, 2013, [5] Takahashi *et al*., 1989, [6] Pique *et al*., 1995, [7] Howarth *et al*., 1981, [8] Stratford and Plaskitt, 1988, [9] Franck *et al*., 1980, [10] Chenault and Melcher, 1993a, [11] Vaden and Melcher, 1990, [12] Geri *et al*., 2004, [13] Shalla *et al*., 1980, [14] Gardner *et al*., 1981, [15] Chenault and Melcher, 1993b, [16] Daubert and Routh, 1990, [17] Chenault *et al*., 1992, [18] Volovitch and Modjtahedi 1990, [19] Qiu and Schoelz, 1992, [20] Wintermantel *et al*., 1993

Table S1. Continued.

Isolate	Original host	Common name	Location (city, district)	Year of collection	Reference	Sequenced region or Accession no.
Turkey						
TUR1	*B. oleracea* var. *botrytis*	Cauliflower	Canakkale, Marmora	2006	this study	AB863166 (Full)
TUR2	*B. oleracea*	Cabbage	Balikesir, Marmora	2006	this study	AB863167 (Full)
TUR4	*R. sativus*	Radish	Balikesir, Marmora	2006	this study	AB863168 (Full)
TUR5	*B. oleracea*	Cabbage	Canakkale, Marmora	2006	this study	AB863169 (Full)
TUR12	*B. oleracea*	Cabbage	Konya, Center Anatolia	2007	this study	AB863170 (Full)
TUR34	*B. oleracea*	Cabbage	Aksaray, Center Anatolia	2007	this study	AB863171 (Full)
TUR50	*B. oleracea*	Cabbage	Nigde, Center Anatolia	2007	this study	AB863172 (Full)
TUR59	*B. oleracea*	Cabbage	Bursa, Marmora	2007	this study	AB863173 (Full)
TUR69	*B. oleracea* var. *botrytis*	Cauliflower	Bursa, Marmora	2007	this study	AB863174 (Full)
TUR81	*B. oleracea*	Cabbage	Bursa, Marmora	2007	this study	AB863175 (Full)
TUR84	*B. oleracea*	Cabbage	Bursa, Marmora	2007	this study	AB863176 (Full)
TUR94	*B. oleracea*	Cabbage	Canakkale, Marmora	2007	this study	AB863177 (Full)
TUR213	*B. oleracea* var. *botrytis*	Cauliflower	Izmir, Aegean	2007	this study	AB863178 (Full)
TUR214	*B. oleracea* var. *botrytis*	Cauliflower	Izmir, Aegean	2007	this study	AB863179 (Full)
TUR216	*B. oleracea* var. *botrytis*	Cauliflower	Izmir, Aegean	2007	this study	AB863180 (Full)
TUR220	*B. oleracea* var. *botrytis*	Cauliflower	Aydin, Aegean	2007	this study	AB863181 (Full)
TUR239	*B. oleracea* var. *botrytis*	Cauliflower	Canakkale, Marmora	2007	this study	AB863182 (Full)
TUR244	*B. oleracea* var. *botrytis*	Cauliflower	Balikesir, Marmora	2007	this study	AB863183 (Full)
TUR249	*B. oleracea* var. *botrytis*	Cauliflower	Balikesir, Marmora	2007	this study	AB863184 (Full)
TUR263	*B. oleracea* var. *botrytis*	Cauliflower	Canakkale, Marmora	2007	this study	AB863185 (Full)
TUR278	*B. oleracea* var. *botrytis*	Cauliflower	Balikesir, Marmora	2007	this study	AB863186 (Full)
TUR279	*B. oleracea* var. *botrytis*	Cauliflower	Balikesir, Marmora	2007	this study	AB863187 (Full)
TUR285	*B. oleracea* var. *botrytis*	Cauliflower	Bursa, Marmora	2007	this study	AB863188 (Full)
TUR289	*B. oleracea* var. *italica*	Broccoli	Bursa, Marmora	2007	this study	AB863189 (Full)
TUR303	*B. oleracea* var. *botrytis*	Cauliflower	Umurbey, Tekirdag	2008	this study	AB863190 (Full)
TUR306	*B. oleracea* var. *botrytis*	Cauliflower	-, Center Anatolia	2008	this study	AB863191 (Full)
Europe						
Croatia						
CRO180A	*B. napus*	Oilseed rape	Zagreb, -	2009	this study	AB863192 (Full)
France						
B29	*B. oleracea* var. *botrytis*	Cauliflower	Rennes, Ille-et-Vilaine	Not known	[6]	X79465 (Full)
Greece						
GRC83	*B. oleracea* var. *italica*	Broccoli	Dytiko, Pella	2008	this study	AB863193 (Full)
GRC84B	*B. oleracea* var. *italica*	Broccoli	Dytiko, Pella	2008	this study	AB863194 (Full)
GRC86B	*R. sativus*	Radish	Athyra, Pella	2008	this study	AB863195 (Full)
GRC86D	*R. sativus*	Radish	Athyra, Pella	2008	this study	AB863196 (Full)
GRC87E	*B. oleracea* var. *botrytis*	Cauliflower	Ionia, Thessaloniki	2008	this study	AB863197 (Full)
GRC87G	*B. oleracea* var. *botrytis*	Cauliflower	Ionia, Thessaloniki	2008	this study	AB863198 (Full)
GRC91B	*B. oleracea* var. *botrytis*	Cauliflower	Epanomi, Thessaloniki	2008	this study	AB863199 (Full)
GRC92A	*B. oleracea* var. *italica*	Broccoli	Epanomi, Thessaloniki	2008	this study	AB863200 (Full)
GRC92C	*B. oleracea* var. *italica*	Broccoli	Epanomi, Thessaloniki	2008	this study	AB863201 (Full)
GRC92D	*B. oleracea* var. *botrytis*	Cauliflower	Epanomi, Thessaloniki	2008	this study	AB863202 (Full)
Hungary						
D/H	*B. oleracea*	Cabbage	Budapest, Budapest	Not known	[7]	M10376 (Full)
Italy						
Bari 1	*Brassica campestris-rapa* L	Perennial wallrocket	Bari, Puglia	Not known	[8]	D00335 (ORF VI)
Cabbage S	*B. ruvo*	Ruvo kale	Bari, Puglia	Not known	[9]	V00141 (Full)
North America						
U. S. A						
BBC	*B. rapa* var *chinensis*	Pak Choi	-, California	1988	[10]	M90542 (Full)
Cabb B-JI	*Brassica* sp.		-, Wisconsin	Not known	[11], [12]	DQ211685 (ORF VI), M32813 (ORF III)
Campbell	*B. oleracea* var. *botrytis*	Cauliflower	-, California	Not known	[13]	M17415 (ORF I)
CM1841	*Brassica* sp.	Turnip	-, California	1967	[14]	V00140 (Full)
CMV-1	Not known		-, California	Not known	[15]	M90543 (Full)
D4	*B. rapa*	Turnip	-, California	Not known	[16]	M23620 (ORF VI)
NY8153	*B. oleracea* var. *botrytis*	Cauliflower	-, New York	Not known	[17]	M90541 (Full)
PV147	*B. rapa*	Not known	-, Wisconsin	Not known	[18]	M37581 (ORF II) X53860 (ORF VI)
South America						
Argentina						
W260	Not known		-, Mendoza	1985	[19], [20]	M94887 (ORF I, ORF VII), L09053 (ORF VI), JF809616 (Full)

Table S2. Detailed results from BEAST analyses of *Cauliflower mosaic virus.*

Model		Marginal likelihood	Bayes factor	TMRCA (95% HPD lower-upper)	Substitution Rate (subs/site/year)	95% HPD Rate-lower (subs/site/year)	95% HPD Rate-upper (subs/site/year)	Population Size	95% HPD Population Size (lower, upper)	Population Growth Rate	95% HDP Growth Rate (lower, upper)
ORFs I-V											
Strict clock	Constant Size	-25503.665	-	2052 (457 - 4697)	4.25×10^{-5}	2.45×10^{-6}	7.81×10^{-5}	1.16×10^{4}	2.24×10^{3}, 2.69×10^{4}	N/A	N/A
	Expansion Growth	-25503.434	1.26E+00	1163 (440 - 2298)	5.18×10^{-5}	1.39×10^{-5}	8.95×10^{-5}	2.73×10^{4}	6.94×10^{3}, 5.92×10^{4}	8.01×10^{-3}	1.30×10^{-3}, 1.46×10^{-2}
	Exponential Growth	-25503.301	1.44E+00	1057 (433 - 2086)	5.28×10^{-5}	1.67×10^{-5}	8.76×10^{-5}	2.04×10^{4}	6.60×10^{3}, 4.22×10^{4}	6.41×10^{-3}	1.92×10^{-3}, 1.09×10^{-2}
	Bayesian Skyline	-25503.692	9.74E-01	4383 (359 - 10271)	3.53×10^{-5}	3.39×10^{-8}	7.29×10^{-5}	3.05×10^{4}	3.02×10^{3}, 7.70×10^{4}	N/A	N/A
Relaxed Exponential	Constant Size	-25410.037	4.59E+40	2998 (71 - 5677)	2.48×10^{-4}	1.48×10^{-8}	6.95×10^{-4}	7.75×10^{3}	1.16×10^{2}, 1.13×10^{4}	N/A	N/A
	Expansion Growth	-25411.092	1.60E+40	634 (108 - 1687)	1.69×10^{-4}	9.62×10^{-6}	3.72×10^{-4}	1.41×10^{4}	1.01×10^{3}, 3.92×10^{4}	2.23×10^{-2}	1.30×10^{-3}, 4.87×10^{-2}
	Exponential Growth	-25404.395	1.30E+43	491 (86 - 1260)	1.71×10^{-4}	1.45×10^{-5}	3.87×10^{-4}	1.24×10^{4}	5.25×10^{2}, 3.40×10^{4}	1.69×10^{-2}	1.66×10^{-3}, 3.70×10^{-2}
	Bayesian Skyline	-25409.932	5.10E+40	1438 (77 - 2822)	1.75×10^{-4}	1.92×10^{-7}	4.29×10^{-4}	8.95×10^{4}	4.50×10^{1}, 1.99×10^{4}	N/A	N/A
Relaxed Lognormal	Constant Size	-25412.128	5.67E+39	2649 (216 - 6083)	5.37×10^{-5}	1.24×10^{-7}	1.14×10^{-4}	1.26×10^{4}	9.14×10^{2}, 2.91×10^{4}	N/A	N/A
	Expansion Growth	-25411.496	1.07E+40	904 (245 - 1920)	7.45×10^{-5}	1.56×10^{-5}	1.38×10^{-4}	1.96×10^{4}	4.04×10^{3}, 4.43×10^{4}	1.04×10^{-2}	1.72×10^{-3}, 2.08×10^{-2}
	Exponential Growth	-25411.528	1.03E+40	848 (274 - 740)	7.21×10^{-5}	1.59×10^{-5}	1.31×10^{-4}	1.77×10^{4}	4.31×10^{3}, 3.85×10^{4}	8.33×10^{-3}	1.22×10^{-3}, 1.55×10^{-2}
	Bayesian Skyline	-25413.313	1.73E+39	3565 (217 - 9665)	5.62×10^{-5}	3.38×10^{-8}	1.28×10^{-4}	2.38×10^{4}	1.48×10^{2}, 6.60×10^{4}	N/A	N/A
ORF VI											
Strict clock	Constant Size	-7092.584	1.20E+00	1687 (784 - 2875)	1.20×10^{-4}	5.35×10^{-5}	1.94×10^{-4}	1.08×10^{3}	4.52×10^{2}, 1.94×103	N/A	N/A
	Expansion Growth	-7090.175	1.35E+01	2179 (886 - 4086)	9.51×10^{-5}	3.31×10^{-5}	1.59×10^{-4}	4.10×10^{3}	8.73×10^{2}, 4.09×10^{3}	3.15×10^{-2}	7.97×10^{-3}, 5.89×10^{-2}
	Exponential Growth	-7092.774	-	1489 (738 - 2447)	1.28×10^{-4}	6.27×10^{-5}	2.00×10^{-4}	1.04×10^{3}	4.61×10^{2}, 1.80×10^{3}	1.01×10^{-3}	4.00×10^{-4}, 2.52×10^{-3}
	Bayesian Skyline	-7090.555	9.20E+00	1911 (781 - 3698)	1.08×10^{-4}	3.44×10^{-5}	1.79×10^{-4}	1.50×10^{3}	7.50×10^{1}, 3.98×10^{3}	N/A	N/A
Relaxed Exponential	Constant Size	-6992.577	3.28E+43	431 (113 - 886)	5.81×10^{-4}	2.47×10^{-4}	9.47×10^{-4}	2.16×10^{2}	8.70×10^{1}, 3.94×10^{2}	N/A	N/A
	Expansion Growth	-6992.859	2.47E+43	404 (101 - 823)	4.91×10^{-4}	1.96×10^{-4}	8.41×10^{-4}	4.14×10^{2}	9.20×10^{1}, 1.03×10^{3}	3.06×10^{-2}	9.14×10^{-8}, 8.16×10^{-2}
	Exponential Growth	-6992.439	3.76E+43	292 (113 - 565)	5.81×10^{-4}	2.32×10^{-4}	9.47×10^{-4}	2.51×10^{2}	8.80×10^{1}, 4.78×10^{2}	5.66×10^{-3}	3.20×10^{-3}, 1.59×10^{-2}
	Bayesian Skyline	-6993.657	1.11E+43	447 (106 - 931)	4.93×10^{-4}	1.87×10^{-4}	8.64×10^{-4}	3.20×10^{2}	1.30×10^{1}, 8.47×10^{2}	N/A	N/A
Relaxed Lognormal	Constant Size	-7000.845	8.40E+39	533 (96 - 1134)	5.03×10^{-4}	1.66×10^{-4}	9.37×10^{-4}	2.85×10^{2}	8.60×10^{1}, 5.25×10^{2}	N/A	N/A
	Expansion Growth	-7002.750	1.25E+39	645 (111 - 1370)	3.49×10^{-4}	8.72×10^{-5}	7.09×10^{-4}	1.00×10^{3}	9.30×10^{1}, 2.45×10^{3}	3.75×10^{-2}	6.73×10^{-6}, 8.32×10^{-2}
	Exponential Growth	-7000.228	1.56E+40	306 (89 - 672)	4.71×10^{-4}	1.77×10^{-4}	8.37×10^{-4}	3.71×10^{2}	1.15×10^{2}, 6.88×10^{2}	8.14×10^{-3}	1.90×10^{-4}, 2.06×10^{-2}
	Bayesian Skyline	-7006.522	2.88E+37	527 (98 - 1128)	3.86×10^{-4}	1.51×10^{-4}	6.68×10^{-4}	4.10×10^{2}	1.70×10^{1}, 1.06×10^{3}	N/A	N/A

[a] Not applicable

Supporting Information References

1. Xie H, Li S, Li W, Ni S, Li Y (1979) A preliminary study of Cauliflower mosaic virus strain 63-3. Acta Microbiol Sinica 19:52-56.
2. Fang R, Wu X, Bu M, Tian Y, Cai F, et al. (1985) Complete nucleotide sequence of *Cauliflower mosaic virus* (Xinjiang isolate) genomic DNA. Chin J Virol 1: 247-256.
3. Farzadfar S, Ahoonmanesh A, Mosahebi GM, Ohshima K, et al. (2007) Partial Biological and Molecular Characterization of *Cauliflower mosaic virus* Isolates in Iran. Plant Pathol J 6:291-298.
4. Farzadfar S, Pourrahim R (2013) Biological and molecular variation of Iranian *Cauliflower mosaic virus* (CaMV) isolates. Virus Genes 47:347-356.
5. Takahashi H, Shimamoto K, Suzuki M, Ehara Y (1989) DNA sequence of gene VI of cauliflower mosaic virus Japanese strain S (CaMV S-Japan). Nucleic Acids Res 17:7981.
6. Piqué M, Mougeot JL, Geldreich A, Guidasci T, Mesnard JM, et al. (1995) Sequence of a cauliflower mosaic virus strain infecting solanaceous plants. Gene 155:305-306.
7. Howarth AJ, Gardner RC, Messing J, Shepherd RJ (1981) Nucleotide sequence of naturally occurring deletion mutants of Cauliflower mosaic virus. Virology 635:678-685.
8. Stratford R, Plaskitt KA (1988) Molecular properties of Bari 1, a mild strain of Cauliflower mosaic virus. J Gen Virol 69:2375-2386.
9. Franck A, Guilley H, Jonard G, Richards K, Hirth L (1980) Nucleotide sequence of Cauliflower mosaic virus DNA. Cell 21:285-294.
10. Chenault KD, Melcher U (1993) The complete nucleotide sequence of cauliflower mosaic virus isolate BBC. Gene 123:255-257.
11. Vaden VR, Melcher U (1990) Recombination implications sites in Cauliflower

mosaic virus DNAs : for mechanisms of recombination. Virology 726:717-726.

12. Geri C, Love AJ, Cecchini E, Barrett SJ, Laird J, et al. (2004) Arabidopsis mutants that suppress the phenotype induced by transgene-mediated expression of cauliflower mosaic virus (CaMV) gene VI are less susceptible to CaMV-infection and show reduced ethylene sensitivity. Plant Mol Biol 56:111–124.
13. Shalla TA, Shepherd RJ, Petersen LJ (1980) Comparative cytology of nine isolates of Cauliflower mosaic virus. Virology 388:381-388.
14. Gardner RC, Howarth AJ, Hahn P, Brown-luedi M, et al. (1981) The complete nucleotide sequence of an infectious clone of cauliflower mosaic virus by Ml3mp7 shotgun sequencing. Nucleic Acids Res 9:2871–2888.
15. Chenault KD, Melcher U (1993) Cauliflower mosaic virus isolate CMV-1. Plant Physiol 101:1395-1396.
16. Daubert S, Routh G. 1990. Point mutations in Cauliflower mosaic virus gene VI confer host-specific symptom changes. Mol Plant-Microbe Interact 3:341-345.
17. Chenault KD, Steffens DL, Melcher U (1992) Nucleotide sequence of Cauliflower mosaic virus isolate NY8153. Plant Physiol 100:542-545.
18. Volovitch M, Modjtahedi N (1990) DNA sequence of gene VI of cauliflower mosaic virus strain PV147. Nucleic Acids Res 18:5297.
19. Qiu SG, Schoelz JE (1992) Three regions of cauliflower mosaic virus strain W260 are involved in systemic infection of solanaceous hosts. Virology 190:773–782.
20. Wintermantel WM, Anderson EJ, Schoelz JE (1993) Identification of domains within gene VI of cauliflower mosaic virus that influence systemic infection of *Nicotiana bigelovii* in a light-dependent manner. Virology 798:789–798.
